# Immunohistochemical Analysis of CD117 in the Mast Cells of Odontogenic Keratocysts

**DOI:** 10.7759/cureus.67558

**Published:** 2024-08-23

**Authors:** Sujatha Varma, Shameena PM, Plakkil Viswanathan Deepthi, Indu G

**Affiliations:** 1 Department of Oral Pathology and Microbiology, Government Dental College, Thrissur, Thrissur, IND; 2 Department of Oral Pathology and Microbiology, Government Dental College, Kottayam, Kottayam, IND

**Keywords:** tumors, odontogenic cysts, odontogenic keratocyst, cd117, mast cells

## Abstract

Background: Odontogenic lesions contain mast cells (MCs), particularly those with a cystic appearance. Because of their high recurrence rates and aggressive clinical behaviour, odontogenic keratocysts (OKCs) require special treatment. A particular kind of protein called cluster of differentiation (CD) 117/ receptor tyrosine kinase (c-KIT) is present on the surface of many cells. Most hematopoietic cells lose their expression of KIT during the differentiation process, with the exception of MCs, which continue to express KIT throughout their lifetime.

Aim: Using the CD117 immunomarker, this immunohistochemical investigation sought to assess the presence and location of MCs in OKCs and examine the relationship between MC numbers in sporadic, syndromic, and recurrent OKCs.

Methods: The study comprised 30 paraffin-embedded tissue specimens, and a histopathological diagnosis was made from hematoxylin and eosin-stained sections with a thickness of 4-5 µ. Out of 30 specimens, 21 were sporadic, six were recurrent OKCs, and three were syndrome-associated OKCs. CD-117/c-kit rabbit polyclonal primary antibody was used to stain the sections for observing MCs, which were then viewed under a light microscope with a digital camera and a desktop computer with MICAPS software for viewing images.

Result: To compare the number of MCs among OKCs, a one-way ANOVA test was used. Our study revealed that a statistically significant increase in MCs has been observed in the subepithelial and deep connective tissue of recurrent OKC (p < 0.05). However, a comparison of the mean MC value among three OKC subtypes did not reveal any statistically significant differences. An increased mast count was observed in the deep connective tissue layer of syndromic OKC under multiple comparisons.

Conclusion: Our study concluded that MCs were present in increased numbers both in the superficial and deep connective tissue of recurrent OKCs, indicative of their aggressive clinical behaviour. Increased mean MC counts observed in some of the sporadic cases may be an indicator of their chances of recurrence in the future.

## Introduction

The most frequent damaging lesions of the jawbones are most likely cysts and lesions in cystic form. Both in the maxilla and mandible, these pathologic diseases have the potential to cause expansive growth and bone loss. Although the precise process of these lesions' genesis and expansion is yet unknown, it is known that a variety of cell types, including mast cells (MCs), can be involved in these events [1].

MCs can engage in a wide variety of biological functions due to their numerous features. They break down antigens, create cytokines, and release a range of physiological mediators that are either freshly generated (like prostaglandins and leukotrienes) or preformed (like histamine, proteoglycans, and proteases) [2]. MCs can respond to various specific and nonspecific stimuli because of the variety of adhesion molecules, immune response receptors, and other surface molecules they carry. Due to their diverse biological properties, widespread distribution, and advantageous positions close to blood vessels, neurons, inflammatory tissues, and neoplastic foci, they can participate in a wide range of physiological, immunologic, and pathological processes.

Although their precise role in odontogenic lesions is uncertain, MCs are present in these lesions, particularly in those in cystic form [3]. In addition, MCs contribute to the growth and enlargement of odontogenic lesions by degranulating their enzyme content in the proximity of bone tissue present in these lesions, which aids in the process of bone remodeling [4]. Since they generate and release proteolytic enzymes that promote tumor and endothelial cell migration and the release of angiogenic factors from stromal tissue, MCs are essential in the process of remodeling the extracellular matrix in neoplastic changes [1,3,4].

MCs have been seen in odontogenic tumors and the connective tissue wall of odontogenic cysts, particularly in sub-epithelial regions, according to a number of investigations [3]. Odontogenic keratocysts (OKCs) are a condition that requires close monitoring because of their aggressive clinical behavior and high recurrence rates.

The purpose of this research is to use the CD117 immunomarker to clarify the existence and location of MCs in OKCs and to examine the relationship between the MC numbers in sporadic, syndromic, and recurrent OKCs.

## Materials and methods

Tissue specimens (n = 30) of OKCs were collected from the archives of the Oral Pathology Department at the Government Dental College in Kozhikode, Kerala, India. These included sporadic (n = 21), recurrent (n = 6), and syndrome-linked OKC (n = 3) cases. All specimens were formalin-fixed and paraffin-embedded. The Institutional Review Board (IRB) and Institutional Ethics Committee approved the study protocol (IEC no. 221/2021/DCC, dated 05/10/21).

Inclusion and exclusion criteria

This cross-sectional retrospective immunohistochemical study included histopathologically confirmed cases of OKCs. Only specimens preserved in formalin-fixed paraffin-embedded blocks were considered. Recurrences of OKCs occurring within one year of the initial presentation were excluded to avoid confounding factors. In addition, archival specimens stored for over five years were excluded to ensure sample integrity. The retrieval of CD117 antigen was found to be difficult from specimens stored in formalin for a prolonged period, and we excluded those specimens from this study. Incomplete or inadequately preserved specimens were also excluded from the study.

Histopathological diagnoses were confirmed using hematoxylin and eosin-stained sections. Tissue sections of 4-5 µm thickness were prepared. Sections were stained with CD-117/c-kit rabbit polyclonal primary antibody (PathnSitu Biotechnologies, India) to identify MCs. Immunohistochemistry (IHC) was performed according to standardized protocols. CD117 immunostaining positivity was observed as brownish staining in the cytoplasmic granules of MCs.

A light microscope (LABOMED LX 300 Pro Series, India) with a digital camera and MICAPS software was used for capturing and analyzing images. Ten high-power fields (40x magnification) per specimen were evaluated. Five fields were selected from the sub-epithelial zone of the connective tissue and five from the deeper connective tissue. Field selection was based on the consensus between two independent observers to minimize inter-observer variability. MCs exhibiting positive CD117 immunostaining were counted in the 10 selected fields per connective tissue wall of the cyst. The quantification followed standards set forth by Patidar KA et al. [5]. 

The terminology used, such as "superficial field 1," "superficial field 2," and "deep connective tissue of the cyst wall field 1," refers to the specific regions within the tissue samples where MC counts were performed. Superficial fields (1-5) are fields selected from the sub-epithelial zone of the connective tissue adjacent to the epithelial lining of the cyst. Deep connective tissue fields (1-5) are chosen from the deeper parts of the connective tissue wall of the cyst, away from the epithelial lining.

Statistical analysis

Data were analyzed using IBM SPSS Statistics for Windows, Version 26.0 (released 2019, IBM Corp., Armonk, NY). Descriptive statistics, including mean and standard deviation, were calculated for MC counts. Comparisons between different groups (sporadic, recurrent, and syndrome-linked OKCs) were made using ANOVA or Kruskal-Wallis tests as appropriate. A p-value of <0.05 was considered statistically significant. Post-hoc tests were conducted to identify specific group differences when overall significance was found. This rigorous statistical approach ensured the reliability and validity of the study's findings.

## Results

Recurrence cases (n = 6) showed significantly higher mean MC counts across all superficial fields (1-5) compared to sporadic (n = 21) and syndrome-associated OKCs (n = 3). The p-values for all fields (0.002, 0.001, 0.001, 0.002, 0.011) indicate a statistically significant difference among the groups. Recurrence cases consistently exhibited higher MC counts than sporadic and syndrome-associated OKCs in all fields (1-5). Significant p-values (0.011, 0.028, 0.002, 0.002, 0.006) indicate notable differences in MC counts among the groups, with recurrence cases having the highest counts (Tables 1, 2).

**Table 1:** Utilizing one-way ANOVA test, mean mast cell count in the sub-epithelial connective tissue of various OKCs was determined OKC: odontogenic keratocysts

Variables		N	Mean	Standard deviation	p-value
Superficial field 1	Recurrence cases	6	16.5	6.77495	0.002
	Sporadic	21	6.0476	5.81787	
	Syndrome-associated OKC	3	4	3.60555	
	Total	30	7.9333	7.17723	
Superficial field 2	Recurrence cases	6	14.8333	9.53764	0.001
	Sporadic	21	3.8571	4.37362	
	Syndrome-associated OKC	3	3.6667	2.3094	
	Total	30	6.0333	7.01959	
Superficial field 3	Recurrence cases	6	17.8333	12.6557	0.001
	Sporadic	21	4.2381	3.54831	
	Syndrome-associated OKC	3	3	1	
	Total	30	6.8333	8.23401	
Superficial field 4	Recurrence cases	6	14.1667	10.70358	0.002
	Sporadic	21	3.9524	4.05557	
	Syndrome-associated OKC	3	2.6667	1.52753	
	Total	30	5.8667	7.01591	
Superficial field 5	Recurrence cases	6	15.6667	17.48904	0.011
	Sporadic	21	3.5714	3.48671	
	Syndrome-associated OKC	3	4	0	
	Total	30	6.0333	9.22696	

**Table 2:** Utilizing one-way ANOVA test, mean mast cell count in the deep connective tissue of various OKCs was determined OKC: odontogenic keratocysts

Variables		N	Mean	Std. deviation	p-value
Deep connective tissue of the cyst wall field 1	Recurrence cases	6	14	12	0.011
	Sporadic	21	4.3333	4.6188	
	Syndrome-associated OKC	3	3.6667	2.51661	
	Total	30	6.2	7.46671	
Deep connective tissue of the cyst wall field 2	Recurrence cases	6	13	14.3527	0.028
	Sporadic	21	3.7619	3.06439	
	Syndrome-associated OKC	3	6.3333	7.0946	
	Total	30	5.8667	7.69565	
Deep connective tissue of the cyst wall field 3	Recurrence cases	6	15.8333	13.67358	0.002
	Sporadic	21	4.3333	3.74611	
	Syndrome-associated OKC	3	2	1	
	Total	30	6.4	8.09257	
Deep connective tissue of the cyst wall field 4	Recurrence cases	6	14.3333	10.5956	0.002
	Sporadic	21	4.2857	4.37199	
	Syndrome-associated OKC	3	1.6667	1.52753	
	Total	30	6.0333	7.151	
Deep connective tissue of the cyst wall field 5	Recurrence cases	6	10.6667	4.27395	0.006
	Sporadic	21	5.1223	4.7656	
	Syndrome-associated OKC	3	1.3454	1	
	Total	30	5.4567	4.5656	

**Figure 1:**
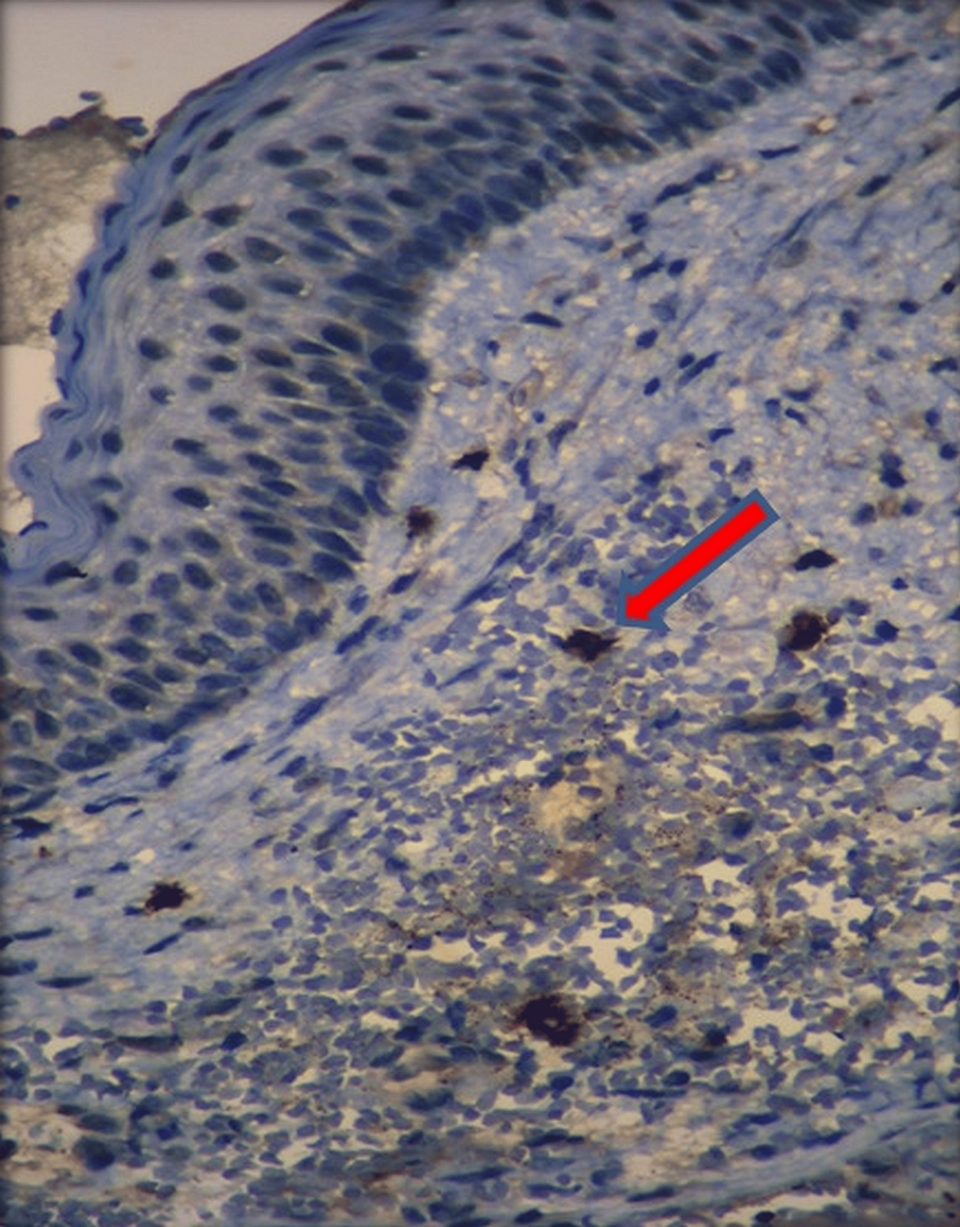
IHC staining: CD 117-positive mast cell within the superficial connective tissue of OKC (x100) The red arrow indicates mast cells. OKC: odontogenic keratocyst

**Figure 2:**
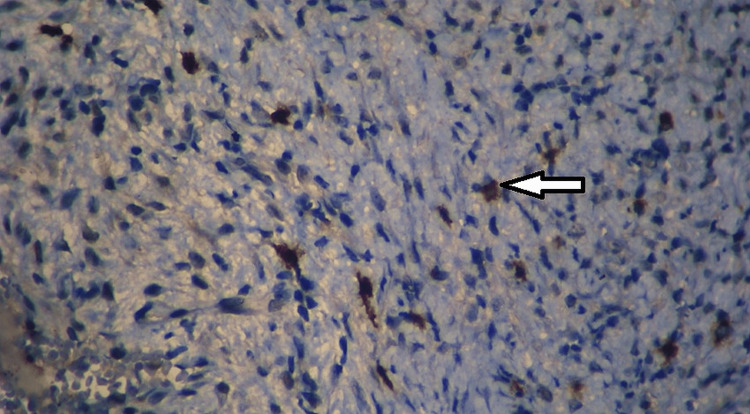
IHC staining: CD117-positive mast cell within the deep connective tissue of OKC (x400) The white arrow indicates the mast cell. OKC: odontogenic keratocyst

The comparisons show no statistically significant differences in MC counts between the two tissue types across all fields, with p-values ranging from 0.46 to 0.948, indicating no significant variation between superficial and deep connective tissue MC counts within the same OKC type (Table 3, Figures 1, 2).

**Table 3:** Group statistics for the comparison

Type	N	Mean	Standard deviation	p-value
Superficial	30	7.9333	7.17723	0.722
Deep connective tissue of the cyst wall	30	6.2	7.46671	
Superficial	30	6.0333	7.01959	0.874
Deep connective tissue of the cyst wall	30	5.8667	7.69565	
Superficial	30	6.8333	8.23401	0.817
Deep connective tissue of the cyst wall	30	6.4	8.09257	
Superficial	30	5.8667	7.01591	0.948
Deep connective tissue of the cyst wall	30	6.0333	7.151	
Superficial				
Deep connective tissue of the cyst wall				
Superficial	30	6.0333	9.22696	0.46
Deep connective tissue of the cyst wall	30	5.5	4.84768	0.5

Table 4 provides multiple comparison results of MC counts in the superficial layer of various OKC types. Recurrence cases had significantly higher MC counts compared to sporadic and syndrome-associated OKCs in all fields. Significant mean differences and p-values (e.g., 0.002, 0.001, 0.000, 0.002, 0.009) support these findings, with recurrence cases showing the largest differences. Table 5 shows multiple comparison results of MC counts in the deep connective tissue layer. Recurrence cases again had significantly higher MC counts than sporadic and syndrome-associated OKCs across all fields. Significant mean differences and p-values (e.g., 0.010, 0.022, 0.003, 0.003, 0.010) indicate the prominence of MC counts in recurrence cases compared to the other OKC types (Tables 4, 5).

**Table 4:** Multiple comparison of mast cells in different OKCs in the superficial layer OKC: odontogenic keratocyst

Dependent variable	Group	Group	Mean difference (I-J)	Standard error	Significance	95% confidence interval	
						Lower bound	Upper bound
Superficial Field 1	Recurrence cases	Sporadic	10.45238	2.72037	.002	3.7074	17.1973
		Syndrome-associated OKC	12.50000	4.15543	.015	2.1969	22.8031
	Sporadic	Recurrence cases	-10.45238	2.72037	.002	-17.1973	-3.7074
		Syndrome-associated OKC	2.04762	3.62716	.840	-6.9456	11.0409
	Syndrome-associated OKC	Recurrence cases	-12.50000	4.15543	.015	-22.8031	-2.1969
		Sporadic	-2.04762	3.62716	.840	-11.0409	6.9456
Superficial Field 2	Recurrence cases	Sporadic	10.97619	2.59436	.001	4.5437	17.4087
		Syndrome-associated OKC	11.16667	3.96296	.024	1.3408	20.9925
	Sporadic	Recurrence cases	-10.97619	2.59436	.001	-17.4087	-4.5437
		Syndrome-associated OKC	.19048	3.45915	.998	-8.3862	8.7672
	Syndrome-associated OKC	Recurrence cases	-11.16667	3.96296	.024	-20.9925	-1.3408
		Sporadic	-.19048	3.45915	.998	-8.7672	8.3862
Superficial Field 3	Recurrence cases	Sporadic	13.59524	2.89313	.000	6.4220	20.7685
		Syndrome-associated OKC	14.83333	4.41932	.006	3.8760	25.7907
	Sporadic	Recurrence cases	-13.59524	2.89313	.000	-20.7685	-6.4220
		Syndrome-associated OKC	1.23810	3.85750	.945	-8.3263	10.8025
	Syndrome-associated OKC	Recurrence cases	-14.83333	4.41932	.006	-25.7907	-3.8760
		Sporadic	-1.23810	3.85750	.945	-10.8025	8.3263
Superficial Field 4	Recurrence cases	Sporadic	10.21429	2.68218	.002	3.5641	16.8645
		Syndrome-associated OKC	11.50000	4.09709	.024	1.3416	21.6584
	Sporadic	Recurrence cases	-10.21429	2.68218	.002	-16.8645	-3.5641
		Syndrome-associated OKC	1.28571	3.57623	.931	-7.5813	10.1527
	Syndrome-associated OKC	Recurrence cases	-11.50000	4.09709	.024	-21.6584	-1.3416
		Sporadic	-1.28571	3.57623	.931	-10.1527	7.5813
Superficial Field 5	Recurrence cases	Sporadic	12.09524	3.75064	.009	2.7958	21.3946
		Syndrome-associated OKC	11.66667	5.72919	.123	-2.5384	25.8717
	Sporadic	Recurrence cases	-12.09524	3.75064	.009	-21.3946	-2.7958
		Syndrome-associated OKC	-.42857	5.00085	.996	-12.8278	11.9706
	Syndrome-associated OKC	Recurrence cases	-11.66667	5.72919	.123	-25.8717	2.5384
		Sporadic	.42857	5.00085	.996	-11.9706	12.8278

**Table 5:** Multiple comparison of mast cells in different OKCs in the deep connective tissue layer OKC: odontogenic keratocyst

Dependent variable	Group	Group	Mean difference (I-J)	Std. error	Sig.	95% confidence interval	
						Lower bound	Upper bound
Deep connective tissue of the cyst wall field 1	Recurrence cases	Sporadic	9.66667	3.03332	.010	2.1458	17.1875
		Syndrome-associated OKC	10.33333	4.63348	.084	-1.1550	21.8217
	Sporadic	Recurrence cases	-9.66667	3.03332	.010	-17.1875	-2.1458
		Syndrome-associated OKC	.66667	4.04443	.985	-9.3612	10.6945
	Syndrome-associated OKC	Recurrence cases	-10.33333	4.63348	.084	-21.8217	1.1550
		Sporadic	-.66667	4.04443	.985	-10.6945	9.3612
Deep connective tissue of the cyst wall field 2	Recurrence cases	Sporadic	9.23810	3.23483	.022	1.2176	17.2586
		Syndrome-associated OKC	6.66667	4.94128	.381	-5.5848	18.9182
	Sporadic	Recurrence cases	-9.23810	3.23483	.022	-17.2586	-1.2176
		Syndrome-associated OKC	-2.57143	4.31310	.823	-13.2654	8.1225
	Syndrome-associated OKC	Recurrence cases	-6.66667	4.94128	.381	-18.9182	5.5848
		Sporadic	2.57143	4.31310	.823	-8.1225	13.2654
Deep connective tissue of the cyst wall field 3	Recurrence cases	Sporadic	11.50000	3.10849	.003	3.7928	19.2072
		Syndrome-associated OKC	13.83333^*^	4.74829	.019	2.0603	25.6063
	Sporadic	Recurrence cases	-11.50000	3.10849	.003	-19.2072	-3.7928
		Syndrome-associated OKC	2.33333	4.14465	.841	-7.9430	12.6097
	Syndrome-associated OKC	Recurrence cases	-13.83333	4.74829	.019	-25.6063	-2.0603
		Sporadic	-2.33333	4.14465	.841	-12.6097	7.9430
Deep connective tissue of the cyst wall field 4	Recurrence cases	Sporadic	10.04762	2.74337	.003	3.2457	16.8496
		Syndrome-associated OKC	12.66667	4.19057	.015	2.2765	23.0568
	Sporadic	Recurrence cases	-10.04762	2.74337	.003	-16.8496	-3.2457
		Syndrome-associated OKC	2.61905	3.65783	.756	-6.4502	11.6883
	Syndrome-associated OKC	Recurrence cases	-12.66667	4.19057	.015	-23.0568	-2.2765
		Sporadic	-2.61905	3.65783	.756	-11.6883	6.4502
Deep connective tissue of the cyst wall field 5	Recurrence cases	Sporadic	6.14286	1.92018	.010	1.3819	10.9038
		Syndrome-associated OKC	8.66667	2.93312	.017	1.3942	15.9391
	Sporadic	Recurrence cases	-6.14286	1.92018	.010	-10.9038	-1.3819
		Syndrome-associated OKC	2.52381	2.56023	.592	-3.8241	8.8717
	Syndrome-associated OKC	Recurrence cases	-8.66667	2.93312	.017	-15.9391	-1.3942
		Sporadic	-2.52381	2.56023	.592	-8.8717	3.8241

## Discussion

Odontogenic cysts and tumors exhibit complicated activity that is dependent on a multitude of biological processes. Numerous investigations have shown that MC degranulation products contribute to the development of cystic lesions by stimulating the production of cytokines and destroying the extracellular matrix, which results in bone resorption and cyst formation.

MCs are essential components in regulating the immune response inside the tumor microenvironment, in addition to being immune effectors. Angiogenesis is controlled by MCs in the early phases of tumor growth, but later on, angiogenesis is taken over by tumor cells [4]. As the tumor progresses, MCs recruit immune cells or suppress antitumor responses [2,4]. MCs cause extracellular matrix degradation by secreting proteolytic enzymes, which facilitate the extracellular matrix's passage into the fluid. Lastly, the action of histamine on smooth muscle contraction and vascular permeability enhances the transudation of serum proteins and contributes to cystic enlargement by raising the intraluminal hydrostatic pressure [5]. In addition, MCs have been linked to the start of prostaglandin production, which can lead to bone resorption and aid in cyst enlargement [5]. MCs and tryptases may contribute to jaw cyst tissue remodeling and the growth of cysts [6].

The synthesis of collagen fibers has also been reported to be influenced by MCs and inflammatory cells, which may help predict the nature, etiology, and biological behavior of periapical lesions [7]. Radicular cysts (RCs) were shown to have a higher mean number of microvasculature and MCs [8]. The idea that MCs and blood vessels contribute to the stromal scaffold of keratocystic odontogenic tumors (KCOTs) is supported by the significant interaction observed between the MC population and microvasculature in KCOTs [9]. MC tryptase and CD34 in KCOT have been shown in certain investigations to positively correlate [10].

MC presence has traditionally been established by histochemical techniques. Toluidine blue (TB) staining is used for this, and it reveals metachromatic granules in the cytoplasm of the cells [5,11]. The majority of MC stains depend on the amount of esterase, heparin, and other glycosaminoglycans present in the cell. It is possible for TB staining to miss young MCs [5]. Several antibodies have been employed in place of histochemical stains to detect human MCs. While MCs from connective tissue were insensitive to formalin fixation, mucosal surface MCs are susceptible to it and do not stain with metachromatic dyes. Tryptase and chymase enzyme profiles in MCs from various anatomical locations have been found to differ [11]. A class of protein called CD117, sometimes referred to as the c-kit and stem cell factor receptor, is present on the surface of various types of cells. MCs, melanocytes, and immature myeloid cells contain larger concentrations of it [12]. Most hematopoietic cells lose their expression of KIT during the differentiation process, with the exception of MCs, which continue to express KIT throughout their lifetime. KIT therefore has a significant impact on the survival, proliferation, and functionality of MCs [4,11].

Numerous investigations have documented MCs in distinct tissue zones within inflammatory lesions, indicating their function in the start, growth, and advancement of these lesions. Some of the studies have shown their increased presence in RCs and OKCs [2,3,5]. Moreover, their presence was marked in syndromic OKCs rather than sporadic OKCs [1,2]. Choudhary et al. found that RCs showed higher MC counts than periapical granulomas [13]. High MC counts were found in RCs and degranulated MCs were abundant in both OKCs and RCs [14]. When odontogenic cysts are implanted in paraffin-embedded tissues, immunohistochemistry (IHC) targeting their contents has been shown to identify more MCs than histochemical staining. Inflamed dentigerous cysts and KCOTs had higher mean MC counts than non-inflamed lesions. Deep connective tissue showed degranulated MC except for noninflamed KCOT analyzed by IHC [15]. The presence of degranulated MCs, perhaps in deeper areas, indicates that these cells are actively growing cystic lesions. According to Teronen et al., there is a greater frequency of degranulated microcysts at the periphery of the cysts near perilesional bone and inflammatory cells. They hypothesized that the involvement of microcysts in the inflammatory process is connected to bone degradation, which in turn causes the enlargement of inflammatory periapical lesions. Tryptase-positive MCs were discovered in the greatest quantity in RCs, while OKCs had the lowest quantity of MCs. Patidar et al. found that OKCs had fewer MCs compared to RCs and dentigerous cysts [5]. The results of these studies vary, and the differences seen among them may be attributed to the methods used to detect MCs and the complex interactions in the tumor microenvironment. 

In the present study, we did not observe an increased MC count in sub-epithelial regions, which is in contrast to the observations by Dos Santos et al. [2]. Studies have shown a significantly increased MC count in syndrome-associated OKCs [2,15]. However, we were unable to ascertain this, probably because of the fewer cases (n = 3) we could obtain during the present study. Sheetal et al. found that MC numbers tend to be greater in lesions of the anterior mandible, and as the age of the patient increases, MC counts decrease, contributing to the pathogenesis of inflammatory lesions [16]. However, we were unable to discover any connection between the patient's age and the number of MCs. In our current investigation, we found that both the deep and superficial layers of recurrent OKCs had mean MC levels that were noticeably high. The higher number of MCs found in sporadic OKCs may be a contributing factor, which signifies their tendency to recur in the future. The clinically aggressive behavior of OKCs could be linked to the presence of increased MC counts in their connective tissue walls. The range of biological activities of MCs depends on the active components like histamines and tryptases, which may cause degradation of the extracellular matrix, thus resulting in cystic expansion. The present study included archival specimens of OKCs, which were preserved in formalin over a period of five years. Therefore, we suggest that the study of MCs in fresh formalin-fixed, paraffin-embedded OKC specimens could yield a better understanding of the correlation between their presence in recurrent lesions. Future therapeutic approaches may be based on confirming the involvement of MCs in the etiology and development of odontogenic cysts.

In the same study, the following points were noted as a limitation of the study as the sample size was relatively small; in cases of syndrome-associated OKCs, there were not many cases, which may limit the generalization of the findings. It is also likely that discrepancies in MC counts seen between the corkscrew or within different studies could stem from differences in the method used for detecting MCs. The failure to detect an elevated tit of MCs in sub-epithelial layers, as opposed to certain pre-existing pieces of research, may point to different methods being used or variations in samples. Moreover, the study also failed to identify any relationship between the age of the patients and the MC count present in them, which could be because of the lack of a diverse cross-section of patients included in the study population. Prolonged storage in the formalin solution was found to impair the immunohistochemical staining, which forced us to exclude some archival specimens. Finally, it is also important to note the cross-sectional study design of the present study, which precludes the ability to define the precise temporal relationships between MC activity and the etiopathogenesis or tumor development of odontogenic cysts and neoplasms.

## Conclusions

MCs were present in increased numbers both in the superficial and deep connective tissue of recurrent OKCs, indicative of their aggressive clinical behavior. Increased mean MC counts observed in some of the sporadic cases may be an indicator of their chances of recurrence in the future. Although we have observed their presence in syndrome-associated OKCs, it was not statistically significant.
